# The morphology and nutrient content drive the leaf carbon capture and economic trait variations in subtropical bamboo forest

**DOI:** 10.3389/fpls.2023.1137487

**Published:** 2023-04-04

**Authors:** Jun Sun, Jinlong Li, Kohei Koyama, Dandan Hu, Quanlin Zhong, Dongliang Cheng

**Affiliations:** ^1^Key Laboratory of Aqueous Environment Protection and Pollution Control of Yangtze River in Anhui of Anhui Provincial Education Department, School of Resources and Environment, Anqing Normal University, Anqing, Anhui, China; ^2^Key Laboratory of Humid Subtropical Eco-geographical Process, Ministry of Education, Institute of Geography, Fujian Normal University, Fuzhou, Fujian, China; ^3^Laboratory of Plant Ecology, Hokkaido University of Education, Asahikawa, Hokkaido, Japan

**Keywords:** bamboo, leaf economic traits, trade-off, subtropical, allometric

## Abstract

Carbon absorption capability and morphological traits are crucial for plant leaf function performance. Here, we investigated the five bamboos at different elevations in Wuyi Mountain to clarify how the leaf trait responds to the elevational gradient and drives the photosynthetic capacity variations. The Standardized Major Axis Regression (SMA) analyses and the Structural Equation Model (SEM) are applied to identify how the bamboo leaf trait, including the ratio of leaf width to length (W/L), leaf mass per area (LMA), photosynthesis rates (Pn), leaf nitrogen, and phosphorus concentration (Leaf N and Leaf P) response to elevation environment, and the driving mechanism of Pn changes. Across the five bamboo species, our results revealed that leaf P scaled isometrically with respect to W/L, leaf N scaled allometrically as the 0.80-power of leaf P, and leaf N and leaf P scaled allometrically to Pn, with the exponents of 0.58 and 0.73, respectively. Besides, the SEM result showed altitude, morphological trait (W/L and LMA), and chemical trait (leaf N and leaf P) could together explain the 44% variations of Pn, with a standard total effect value of 70.0%, 38.5%, 23.6% to leaf P, leaf N, and W/L, respectively. The five bamboo species along the different elevational share an isometric scaling relationship between their leaf P and W/L, providing partial support for the general rule and operating between morphological and chemical traits. More importantly, the leaf W/L and leaf P as the main trait that affects leaf area and P utilization in growth and thus drives bamboo leaf photosynthetic capacity variations in different elevations.

## Introduction

The leaf is the main photosynthetic organ of most vascular plants in the world, which reflects the long-term adaptation characteristics of plants and thus is most sensitive to environmental changes. The field of the leaf economic spectrum shows that there have mechanism trade-offs between acquisition and conservation among the leaf traits, which are closely related to the adjustment of plant life history strategies (Westoby et al., 2002; Reich, 2014). Several previous studies have found that from leaf anatomy structure to leaf morphology (Sack and Scoffoni, 2013; Li et al., 2022a; Shi et al., 2022; Shi et al., 2023), leaf–branch (Sun et al., 2019) and leaf–stem–root dimensions (Li et al., 2021a) reveal that leaf traits play an important role in understanding plant economic spectrum variations. It is also a critical basis for learning the adaptation of forest ecosystem functions to future climate change (Poorter et al., 2009).

The main factors that affect the leaf traits include altitude, light, temperature, humidity, and other abiotic factors. Indeed, light availability is crucial for shaping leaf traits. For instance, prior studies indicate that leaf mass per area (LMA) decreased with decreased light intensity (Ackerly et al., 2002). In a mixed forest community, a decrease in a light gradient along the exposed canopy to the closed canopy resulted in a significant increase in nitrogen concentration per leaf area (*N*_area_) (Okubo et al., 2012) and a decrease in leaf LMA. In addition, the low LMA and not the high LMA of species was always favored in north-facing slopes (Li et al., 2021b). Therefore, to adapt to the low light conditions, the plants try to keep decreasing the structural cost per unit leaf area and increase nitrogen content to strengthen photosynthesis for survival. In both contexts, the elevations not only affect the light availability but also have comprehensive effects on temperature, moisture, and soil nutrient conditions to leaf functional traits (Hulshof et al., 2013). Previous works suggest that the leaf size and thickness of *Rhododendrons* decreased with increasing elevation in the Sikkim Himalaya (Basnett and Devy, 2021), and other studies show that LMA and *N*_area_ have decreased with the increase of elevational gradients (Read et al., 2014). Indeed, altitude was usually found to affect the plant leaves’ morphology traits (Manishimwe et al., 2022) and nitrogen or phosphorus concentration (van de Weg et al., 2009). However, the elevational environments could not independently dominate the adjustment of leaf functional traits, but the scaling relationships between leaf traits might be another reason that strongly affects the leaf function performance. For instance, He et al. (2006b) found that N content (*N*_area_) and the photosynthetic rate (*A*_area_) are significantly positively correlated with LMA in the Tibetan Plateau, which is consistent with Wright et al.’s (2004) test in global data. Previous work indicates that N allocation and Rubisco activation state have a strong influence on photosynthetic rates in forests (Bahar et al., 2017). Interestingly, the isometric scaling relationship between N and P content in three subtropical forests, which is different from results reported by Wright et al. (2004), suggests that leaf P may be defining the photosynthetic capacity in stronger P limitation ecosystems (Chen et al., 2020), especially in the subtropical forest (Zhang et al., 2018). Thus, we spectate a general rule that controls carbon assimilation rate variations and operates based on the scaling of leaf morphology traits and leaf N and P concentration in the subtropical forest along the different elevations.

Unfortunately, although the majority of woody species are examined in these contexts, little is known about the leaf functional traits response to environmental factors among grass species, particularly for large perennial grasses such as bamboo that grow in tropical and temperate forests. With increasing elevation, the ratio of bamboo leaf width to length (W/L) increased significantly while leaf area and mass decreased (Guo et al., 2018). Further, the W/L has been demonstrated to be strongly correlated with leaf shape variations and is critical to learn the scaling relationships between bamboo leaf size and leaf structural, chemical, and physiological traits (Lin et al., 2020; Shi et al., 2020; Yao et al., 2022). Moreover, the specific leaf area of *Fargesia nitida* and *F. angustissima* may be adjusted by the species-specific sensitivity to temperature and show the non-linear changes along the different elevation gradients in Wolong Nature Reserve, West China (Wu et al., 2010). Indeed, altitude was usually found to affect the bamboo leaves’ morphology (i.e., leaf length and leaf width) and chemical traits (i.e., leaf N and leaf P) (Guo et al., 2018; Aribal et al., 2022). Thus, it is necessary to properly consider changes in bamboo leaf morphological characteristics because bamboo leaf morphology has a crucial influence on leaf area and is available for light capture (Guo et al., 2018; Lin et al., 2020). Under the open habitat area, dwarf bamboo (*F. nitida*) leaves might become thinner and wider, and leaf LMA, the dark respiration rate, and light-saturated point decreased, but the leaf chlorophyll content and nitrogen concentration are increased (Yang et al., 2013). In addition, other studies have found that the photosynthetic characteristics of *Phyllostachys edulis* are closely related to the growth stage, and the maximum photosynthetic rate decreases gradually as the leaf matures to senescence, while the light compensation point increases gradually as the leaf senescence (Shi et al., 2009). In the future, bamboo may adapt to nitrogen deposition or drought environments by changing the aboveground and underground nitrogen allocation and leaf N:P ratio (N:P) (Gao et al., 2020). More importantly, recent reports suggest that leaf P plays a crucial role in the high utilization of Moso bamboo growth (Li et al., 2021c; Li et al., 2022b). However, few studies have examined the scaling relationships between bamboo leaf morphological traits and leaf N and P along an altitudinal gradient. Further, the driving mechanism based on leaf morphological traits and leaf N and P that affects the photosynthetic capability is also not well understood.

Leaf traits include LMA, the W/L, nitrogen and phosphorus concentration (leaf N and P), and photosynthesis rates (*P*_n_) collected from five bamboos located along the different elevations in Wuyishan National Park. The data were used to here determine 1) how bamboo leaf LMA and W/L scales to *P*_n_, leaf N, and leaf P across the different elevational gradients, 2) and how these leaf traits together drive the bamboo’s carbon capture capability changes. We first analyzed the bamboo leaf trait variations and their scaling relationships across the different elevational gradients. Then, we established a structural equation model to explore the mechanisms of how the altitude, leaf morphology, and chemical traits drive the carbon assimilation rates to change.

## Materials and methods

### Site description

The samples sites are located in the Wuyishan National Park (117°24’13”–117°59’19”E, 27°31’20”–27°55’49”N) on the border of Fujian and Jiangxi Province. The total area of Wuyishan National Park is approximately 1,280 km^2^, and the Jiangxi area accounts for 278.57 km^2^. The region has a subtropical monsoon climate, with high temperatures and rain in July and pleasantly moist in January. The average annual temperature in the park is approximately 17°C–19°C, and the average annual precipitation is 1,684–1,780 mm. The highest peak of Wuyi Mountain is 2,160.8 m above sea level and the highest peak in the southeast of the Chinese mainland. There are developed complete altitudinal belt spectra in vegetation along the different elevations in Wuyi Mountain. The forest communities including from *P. edulis* forest and evergreen forest in low elevations, coniferous and broad-leaved mixed forest and deciduous forest in medium elevations, to dwarf forest and mountainous steppe in high elevations.

Five typical bamboo species (*P. edulis*, *I. tessellatus*, *O. oedogonatum*, *Yushania hirticaulis*, and *Y. wuyishanensis*) were examined along different elevations (see Sun et al., 2017). Owing to the size differences among the five species, two plot sizes were used. Specifically, three 10 m × 10 m plots were established for *P. edulis* at elevations 840, 1,040, and 1,240 m a.s.l.; three 10 m × 10 m plots were established for *O. oedogonatum* at elevations 1,100, 1,200, and 1,400 m a.s.l.; three 5 m × 5 m plots were established for *I. tessellatu* at elevations 1,040, 1,440, and 1,840 m a.s.l.; and three 5 m × 5 m plots were established for *Y. hirticaulis* (1,800 m a.s.l.) and *Y. wuyishanensis* at an elevation of 2,100 m a.s.l. All plots were located at least 20 m apart.

### Sample collection

In the summer of 2017, three individuals of 3-year-old bamboo with average height and DBH were selected from each plot between 9:00 and 12:00 a.m. Overall, nine branches in each altitude gradient (three branches for each individual and the total number of samples is 99) were collected from canopies and immediately placed in water to reduce water loss. Embolisms were removed by recutting branch ends under water (Yoder et al., 1994; Mori et al., 2010; Michaletz et al., 2016). The LI-6800 portable photosynthesis system (LI-COR, Lincoln, NE, USA) was used to measure *P*_n_. We randomly selected and tested 5 to 10 leaves (no visible signs of herbivory or disease) and recorded the mean values of *P*_n_ for each branch. The light intensity of the leaf chamber is set to 1,600 μmol m^-2^ s^-1^, the temperature of the leaf chamber is set to 25°C, and the humidity is 55%. The carbon dioxide concentration is set to 400 µmol mol^-1^, and the flow rate is set to 500 mmol s^-1^.

When the photosynthetic and respiration measurement was done, the leaves were brought back to the laboratory and scanned by an Epson V19 scanner (Epson V19, Epson, Suwa, Japan). The length and width of leaves were calculated by Image J software (National Institute of Health, Bethesda, ML, USA). The W/L is calculated as the ratio of width to length. Finally, the leaf samples were placed in the oven at 105°C for 1 h and then dried at 75°C to constant and weighed. The LMA was calculated by leaf weight and area. In addition, the dried bamboo leaves were crushed by a ball mill and screened through the 100-mesh nylon mesh and then sealed in a sealed bag for measurements. The leaf nitrogen concentration in leaves (leaf N) were measured with a Germany Vario EL III Element Analyzer, and phosphorus concentration (leaf P) was determined by a continuous flow analyzer (San ++, SKALAR, Netherlands).

### Data analysis

The SPSS19.0 software was used to analyze the mean and standard deviation of the leaf functional traits of each bamboo species, and Pearson correlation analysis analyzed the correlations between traits. The relationships between bamboo leaf functional traits were best fit by the mathematical equation log (*y*) = log (*β*) + *α*log (*x*), where *β* is the normalization constant and *α* is the scaling exponent. Model Type II regression was used to determine the numerical values of *β* and *α* using the (Standardised) Major Axis Estimation package ‘smatr’ version 4.0.0 in R software (R Core Team, 2020; Warton et al., 2012). The data from species showing no statistically significant differences in the numerical values of the two regression parameters were pooled to determine a common scaling exponent using the standardized major axis package in R (Warton et al., 2006; Warton et al., 2012). The significance level for testing slope heterogeneity was *P* < 0.05 (e.g., slope heterogeneity was rejected when *P* > 0.05). When *α* > 1 or *α* < 1, there is an allometric relationship between *Y* and *M*, while, when *α* = 1 or -1, there is an isometric scaling relationship.

A structural equation model was used to create an empirical model for predicting how morphology and nutrient affect the bamboo leaf carbon capture and economic trait variations. The model ran through SPSS AMOS 22.0 (SPSS. Inc. Chicago. IL, USA). The SEM was simplified and evaluated using maximum-likelihood chi-square tests (Grace et al., 2007). The CMIN/DF (the ratio of the chi-square test value and the degrees of freedom) was between 0.0 and 2.0, and CFI ≥ 0.90 or root mean square error of approximation (RMSEA) < 0.08; the model was considered appropriate in this study.

## Results

### Leaf-functional traits of five bamboos in different elevations

The leaf P of *P. edulis* and *O. oedogonatum* was the highest at low elevation, with a value of 1.71 g/kg, while the leaf P of *I. tessellatus* leaves was the highest at high elevation, with the values of 1.46 g/kg. Except for leaf P, no significant differences have been found in the W/L, leaf N, LMA, and *P*_n_ of *P. edulis* among the three elevations. The leaf N and LMA of *O. oedogonatum* were significantly higher at low elevations, but the leaf N of *I. tessellatus* was the highest at high elevations, and there was no significant difference in LMA between the three elevations. The *P*_n_ was the highest at high elevation for *O. oedogonatum* and *I. tessellatus*. The W/L, leaf N, leaf P, LMA, and *P*_n_ in the leaves of *Y. wuyishanensis* were significantly higher than those of *Y. hirticaulis* (T-test, *P* < 0.05).

### The scaling relationships between bamboo leaf traits

**Figure 1:**
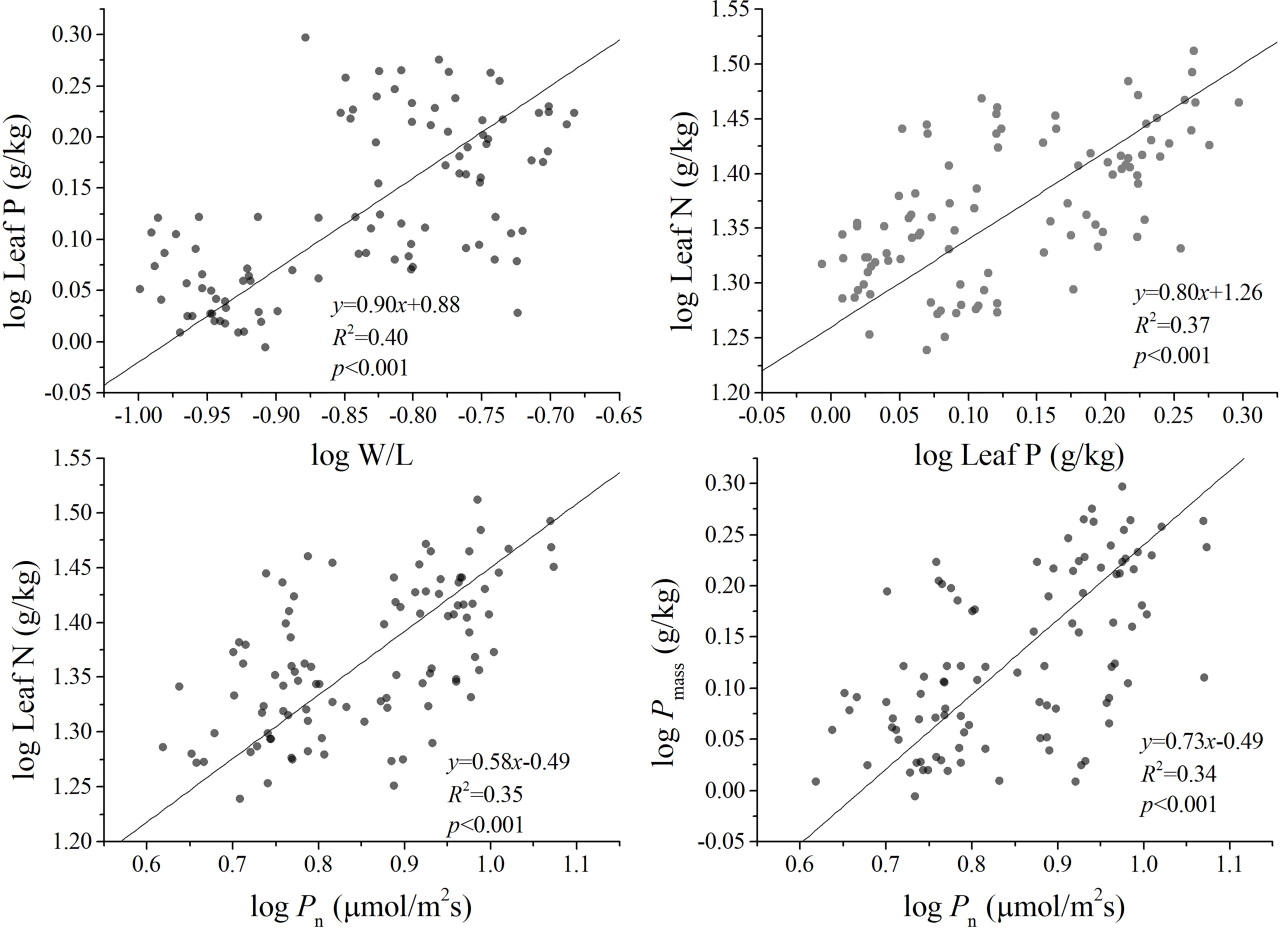
The allometric relationships among the functional traits of five bamboos in Wuyi Mountain. W/L, the ratio of leaf width to length; *P*_n_, photosynthesis rates, leaf N, leaf nitrogen concentration; leaf P, leaf phosphorus concentration.

There are significant positive correlations between W/L and leaf P, LMA versus *P*_n_, leaf N and leaf P vs. *P*_n_, leaf P and LMA vs. *P*_n_, respectively, in bamboo species. The leaf P of different bamboos showed an isometric scaling relationship concerning W/L with *α* = 1.0, leaf N vs. *P*_n_, and leaf P vs. *P*_n_ showed an allometric scaling relationship with exponents of 0.58 and 0.73, respectively. Meanwhile, leaf P and leaf N showed an allometric scaling relationship with an exponent of 0.80 (**Figure 1**). These results indicated that there was a significant trade-off between leaf P and W/L, leaf P and *P*_n_, leaf N and *P*_n_, and leaf N and leaf P.

**Figure 2:**
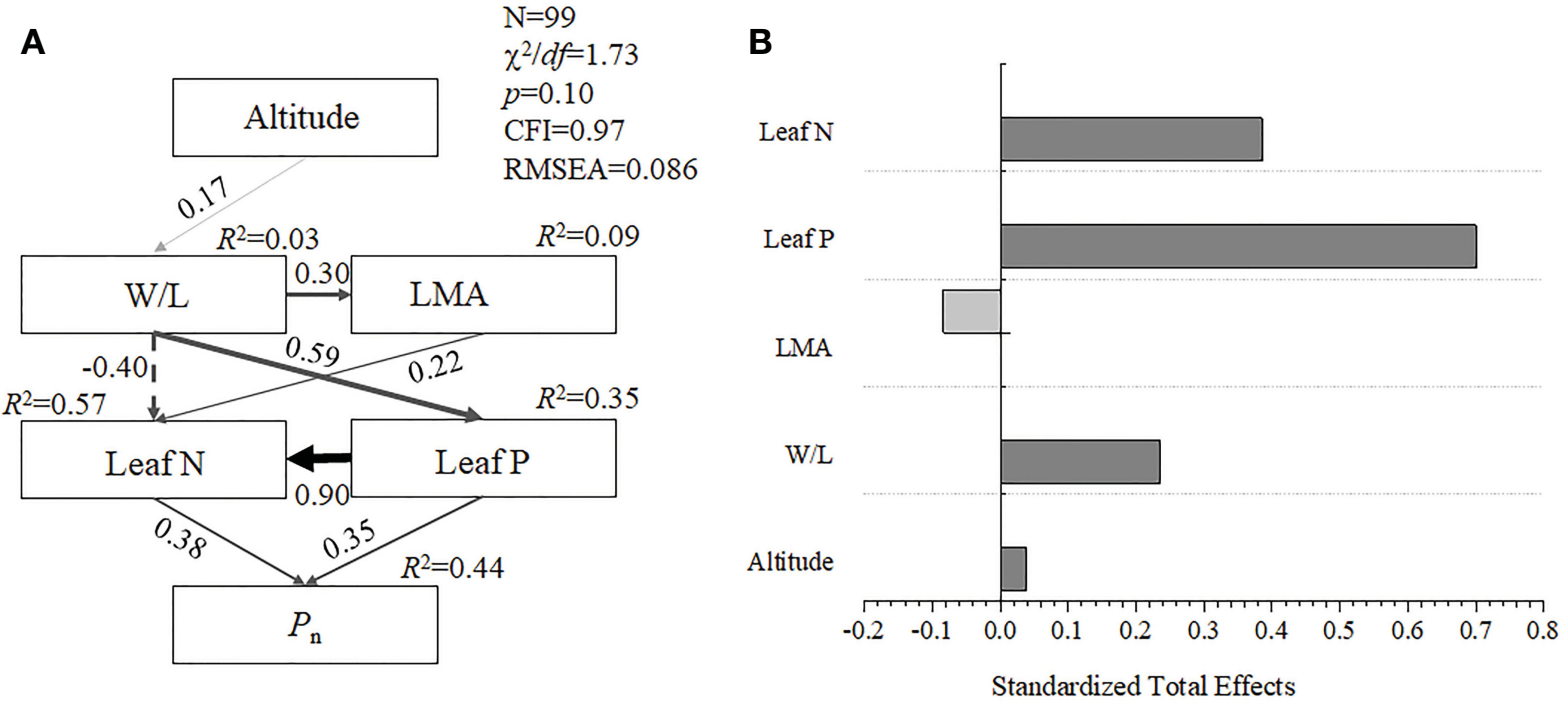
Structural equation model analysis **(A)** and standardized total effects values **(B)** of photosynthesis rates driven by leaf functional traits in five bamboos, Wuyi mountain. W/L, the ratio of leaf width to length; LMA, leaf mass per area; Pn, photosynthesis rates; Leaf N, leaf nitrogen concentration, Leaf P, leaf phosphorus concentration.

The structural equation model showed that elevation influenced LMA by regulating the W/L of bamboo leaves, and leaf nitrogen and phosphorus concentration drove the changes in photosynthetic characteristics. This model had a high goodness of fit (χ^2^/*df* = 1.73, CFI = 0.97, RMSEA = 0.086). The altitude, W/L, LMA, and leaf N and P could together explain 44% of the *P*_n_ variation in five bamboo species (**Figure 2A**). Among them, the standard total effect value of leaf P is 70.0%, leaf N is 38.5%, and W/L is 23.6% (**Figure 2B**). These results indicated that the leaf W/L rather than LMA mainly (<0.001) affects the leaf N and *P* and thus drive the changes of *P*_n_ among five bamboos at the different elevations.

## Discussion

### The response of bamboo leaf functional traits to different elevations

The leaf P of *P. edulis* and *O. oedogonatum* decreased significantly with increasing elevations. It is consistent with the result that Huang et al. (2020) found the leaf P of *P. edulis* leaves decreased with increasing elevations. Thus, our results show the limitation of phosphorus to *P. edulis* and *O. oedogonatum* growth enhanced with the altitude increasing. A potential cause for such a low leaf P may be the temperature decreased with increasing elevations, which inhibited the soil phosphorus mineralization ability (Zhang et al., 2022). The second reason is bamboo renowned for its fast growth rate (Song et al., 2017), and fast-growing organisms need to increase demand for P content that constituted ribosomes, ATP, and RuBisCO (Reich and Oleksyn, 2004; Li et al., 2021c). Owing to environmental stresses increased with increased elevations (i.e., low temperatures and nutrient loss), the photosynthetic ability and growth rates will decrease to some extent, together resulting in a decrease in leaf P storage. In contrast, the leaf P of *I. tessellatus* showed higher at the high-elevation site than at either the low or middle elevation (**Table 1**), which concurred with Han et al.’s (2005) report that leaf P increased with the mean annual temperature across China. Interestingly, previous studies demonstrated that precipitation plays a crucial role in limiting the distribution of Moso bamboo rather than the temperature across mainland in China (Shi et al., 2020). Thus, not only climate factors but also the difference in vegetation types, soil physical traits, and chemical properties affected the leaf P and may exert huge differences (van de Weg et al., 2009). Likewise, the leaf N of *I. tessellatus* and *O. oedogonatum* showed opposite trends with elevation changes (**Table 1**). The controversial exerts herein indicate that leaf N may not be sensitive to temperature changes. Indeed, Hong et al. (2014) reported that no relationship has been found between the leaf N concentration and the annual average temperature. These results suggest that the elevation gradient has a stronger regulation on the leaf functional traits of *O. oedogonatum* and *I. tessellatus* than *P. edulis*. On the other hand, in the understory habitat, the responses of leaf N and P to the elevation gradient were significantly different between *O. oedogonatum* and *I. tessellatus* (**Table 1**), indicating that light availability is another factor that affects these traits. Furthermore, another work suggests that the variation of phylogenetics was the main factor that affected the leaf N concentrations rather than temperature (He et al., 2006a). Generally, leaf *P* and N are among the most crucial nutrients and limit photosynthesis ability in terrestrial ecosystems (Elser et al., 2007). Our results indicated that *P*_n_ was the highest at high elevations for *I. tessellatus* might benefit by having a higher nutrient concentration (leaf P the leaf N) than lower and medium elevations (**Table 1**).

**Table 1:** The functional traits of five bamboo leaves.

Species	Altitude (m)	W/L	Leaf N (g kg^-1^)	Leaf P (g kg^-1^)	LMA (g cm^-2^)	*P*_n_ (μmol m^-2^·s^-1^)
*P. edulis*	840	0.16 ± 0.01a	26.29 ± 1.71a	1.71 ± 0.12a	51.97 ± 2.78a	9.28 ± 1.14a
	1,040	0.15 ± 0.02a	25.75 ± 3.89a	1.63 ± 0.29b	51.65 ± 3.74a	9.41 ± 1.22a
	1,240	0.16 ± 0.02a	28.31 ± 2.01a	1.46 ± 0.20b	49.95 ± 3.46a	9.25 ± 1.05a
	ALL	0.15 ± 0.02B	26.78 ± 2.84A	1.59 ± 0.23A	51.19 ± 3.34C	9.31 ± 1.10A
O. oedogonatum*	1,100	0.12 ± 0.01a	26.41 ± 2.16a	1.21 ± 0.09a	50.71 ± 1.34a	5.88 ± 0.85ab
	1,200	0.12 ± 0.004a	20.74 ± 1.20b	1.08 ± 0.05b	43.93 ± 4.05b	5.68 ± 1.07b
	1,400	0.11 ± 0.01a	21.48 ± 0.72b	1.05 ± 0.04b	45.43 ± 3.20b	6.68 ± 1.21a
	ALL	0.12 ± 0.008C	22.88 ± 2.93B	1.11 ± 0.09C	46.69 ± 4.19D	6.08 ± 1.10B
I. tessellatus*	1,040	0.18 ± 0.01a	19.23 ± 1.16b	1.28 ± 0.16b	55.83 ± 3.79a	5.48 ± 0.60b
	1,440	0.16 ± 0.01b	19.29 ± 1.43b	1.33 ± 0.21b	59.08 ± 4.18a	6.57 ± 1.69ab
	1,840	0.19 ± 0.01a	23.96 ± 2.39a	1.60 ± 0.10a	58.90 ± 3.16a	6.89 ± 1.35a
	ALL	0.18 ± 0.12A	20.83 ± 2.81C	1.40 ± 0.22B	57.94 ± 3.90A	6.31 ± 1.39B
Y. hirticaulis*	1,740	0.11 ± 0.004C	22.48 ± 1.37BC	1.18 ± 0.08C	54.36 ± 3.19B	6.85 ± 1.88B
*Y. wuyishanensis*	2,100	0.18 ± 0.01A	24.77 ± 3.30A	1.53 ± 0.13AB	42.04 ± 1.85E	8.71 ± 1.21A

Data in the table are mean ± standard deviation, N = 9 in each altitude gradient, the *indicating the habitat in the understory. Capital letters indicate the comparison between bamboos, and lowercase letters indicate the comparison between the elevations to same bamboos. Different letters represent significant differences at P < 0.05, and same letters indicate no significant difference. W/L, the ratio of leaf width to length; LMA, leaf mass per area; P_n_, photosynthesis rates, leaf N, leaf nitrogen concentration; leaf P, leaf phosphorus concentration.

### Leaf phosphorus concentration and the ratio of width to length control the scaling of bamboo leaf traits

The functional traits of bamboo leaves may not only be regulated by a single environmental factor but are also constrained by the scaling relationships between leaf traits. Previous studies have found that there are extensive resource allocation strategies between leaf economic traits in plants, including leaf N and P, *P*_n_, the respiration rate (*R*_d_), and the leaf life span (LL) and LMA (Wright et al., 2004; Li et al., 2021a). For instance, Reich et al. (2010) suggest that a two-thirds allometric relationship exists between leaf N and P in major global plant communities. Indeed, our results show a significant positive correlation between leaf N and P across five bamboos (**Table 2**) with the scaling exponents of 0.80 (CIs: 0.68–0.94) (**Figure 1**). Moreover, it is not significantly different from the exponents of 0.69 (CIs: 0.63–0.79) that were found between leaf N and P in the global data (*P*_0.69_ = 0.06) reported by Reich et al. (2010). The scaling relationship between leaf N and P and *P*_n_ was *α* < 1.0, with the exponents of 0.58 and 0.73, respectively (**Figure 1**). It indicates that more leaf P than N might be needed to increase the *P*_n_. There may be two reasons. One is that plants tend to have closer trait correlations when facing the pressure of resource acquisition and utilization (Liu et al., 2019). Under the condition of general phosphorus deficiency in a subtropical forest, the photosynthetic carbon acquisition capacity of leaves is limited by the change in leaf phosphorus content, indicating that bamboos have a high demand for the limiting the element phosphorus (Li et al., 2021c; Aribal et al., 2022). Second, the distribution of phosphorus components in leaves leads to the possibility that bamboo allocates more leaf P to the photosynthetic system. The plants usually increase metabolic phosphorus and reduce phospholipid input to enhance photosynthetic phosphorus utilization efficiency adapted to low-phosphorus environments (Hidaka and Kitayama, 2013; Hayes et al., 2018). Previous studies also indicate that bamboo could maintain the normal photosynthesis process and growth under low-phosphorus conditions (long-term nitrogen deposition test) (Song et al., 2020).

**Table 2:** Correlation between leaf functional traits of five bamboo species in Wuyi Mountain.

Traits	W/L	Leaf N	Leaf P	LMA	*P*_n_
W/L	1				
Leaf N	0.07	1			
Leaf P	0.59**	0.62**	1		
LMA	0.30**	-0.16	0.21*	1	
*P*_n_	0.21*	0.60**	0.59**	-0.18	1

* indicates significant correlation at the 0.05 level; ** indicates a highly significant correlation at the 0.01 level. W/L, the ratio of leaf width to length; LMA, leaf mass per area; P_n_, photosynthesis rates, leaf N, leaf nitrogen concentration; leaf P, leaf phosphorus concentration.

As a crucial leaf trait, the LMA can be seen as the leaf investment of plants and closely correlates with the leaf life span (LL), nutrient concentration, and photosynthetic capacity (Westoby et al., 2002). In this study, LMA is positively related to leaf P but not with *P*_n_ (**Table 2**). Moreover, W/L is positively correlated with leaf P and *P*_n_, respectively (**Table 2**), which indicates that *P*_n_ keeps pace with a leaf shape change. Consistent with some prior reports (e.g., Shi et al., 2020; Du et al., 2021), bamboo leaf surface area or specific leaf area mainly relies on the variation of W/L and thus has a closer relationship with carbon capture capacity. Therefore, we see that the positive relationship between *P*_n_ and W/L is an important attempt to reveal the links between bamboo physiological traits and leaf shape changes. Our results are consistent with the Lin et al.’s (2020) reports that W/L plays a key role in the studied leaf functional traits in bamboo, particularly in the scaling exponent of leaf dry mass vs. leaf area. Such a scaling relationship is often referred to as “diminishing returns” (Sun et al., 2017), indicating that gains in leaf area do not keep pace with increasing leaf mass investments. Nevertheless, our results found the isometric relationship between leaf P and W/L, which indicated that bamboo leaf shape increased proportionately with phosphorus concentration increase. Further, the allometric scaling relationship has been found between *P*_n_ and leaf P. In this context, it is critical to note that *P*_n_ gains do not keep pace with increasing leaf P and W/L investments. When these patterns are concerned together, it is reasonable to conclude that W/L should be included in the study of the leaf economic spectrum for bamboo in the future.

### Effects of bamboo leaf functional traits on photosynthesis capability

Since the adjustment of leaf economic traits is deeply correlated with photosynthesis and productivity, it is necessary to quantitatively analyze the processes of leaf economic traits driven by environmental and intrinsic factors (Li et al., 2021a). The structural equation model has been used to quantitatively analyze the causal relationship between variables, which has been more mature in the field of plant functional trait research (Vile et al., 2006; Yu et al., 2022). This study hypothesized that elevation drives changes in leaf morphological characteristics and nutrient content that affect photosynthesis (**Figure 2**) as an initial model. Indeed, the data analysis revealed that altitude, morphology trait (LMA and W/L), and nutrient content (leaf N and P) together explain 44% variations of *P*_n_ (**Figure 2**). On the one hand, compared with leaf W/L, the mean value of the LMA ranged from 42.04 g cm^-2^ to 59.08 g cm^-2^ and the coefficient of variation of LMA among five bamboos is lower (i.e., LMA is 12.26% and W/L is 20.64%). Previous studies have also found that W/L performed a higher variability than LMA (Lin et al., 2020). Therefore, contrary to W/L, the response of LMA to the elevational gradient might not be more sensitive than W/L. These results have also confirmed the “diminishing returns” hypothesis, indicating that there exists a constant allometric relationship between leaf bamboo area (carbon capture) and dry mass (investment) in different environments (Sun et al., 2017; Lin et al., 2020). On the other hand, tropical and subtropical forests are usually phosphorus-limited ecosystems (Hou et al., 2020), and other studies showed that, in habitats with high resource acquisition and utilization pressure, plant functional traits will have closer correlation characteristics (Liu et al., 2019). Therefore, in the structural equation model of this study, the standard direct effect contribution of leaf P to the model is 70.0%, which also proved that bamboo species have the characteristics of high demand for phosphorus concentration in a subtropical forest (Li et al., 2021c; Li et al., 2022b). Furthermore, we highlight that future studies need to focus on the leaf phosphorus concentration and its relationship with leaf shape traits, particularly W/L, which may provide an important view to study bamboo leaf economic traits changes in different environments.

## Conclusion

Our results documented detailed information on leaf LMA, W/L, leaf N and P concentrations, and the *P*_n_ of five bamboo species along the altitudinal environment in Wuyi Mountain. Especially, the leaf P of *P. edulis* and *O. oedogonatum* decreased with increasing elevation supporting the hypothesis that leaf P may be controlled by temperature. However, the different trends of leaf N along the elevation gradient between *I. tessellatus* and *O. oedogonatum* indicated that leaf N is not sensitive to temperature. The scaling exponent of leaf N vs. leaf P across the five bamboo species is not different from the two-thirds power law and consistent with scaling in the major global forest. In addition, the isometric relationship between leaf P and leaf W/L suggests a general rule that performs in leaf morphological and chemical traits and is insensitive to elevations and species. Compared with leaf N and LMA, the structural equation model also showed the leaf W/L and leaf P as the main trait that drives the leaf photosynthetic capacity variation in different elevational environments. In sum, our findings confirmed that elevation is not the single factor that controls the variations of leaf functional traits of five bamboo species in Wuyi Mountain. More importantly, the scaling between morphology and nutrient content leads to reduce the ability of the elevation environment to shape the bamboo leaves but defines the carbon assimilation rates.

## Data availability statement

The original contributions presented in the study are included in the article/**Supplementary Material**. Further inquiries can be directed to the corresponding author.

## Author contributions

JS and DC conceived and designed the experiments. JS and QZ performed the experiments. JS, JL and DH analyzed the data. JS and DC wrote and KK revised the manuscript. All authors contributed to the article and approved the submitted version.
