# Supplementary material for: The morphology and nutrient content drive the leaf carbon capture and economic trait variations in subtropical bamboo forest
Source: Front Plant Sci. 2023 Apr 4;14:1137487. doi: 10.3389/fpls.2023.1137487 (PMC10110945; doi:10.3389/fpls.2023.1137487)
Supplement: Supplementary file 1 [file DataSheet_1.pdf]

Supplementary Material

The morphology and nutrient content drive the leaf carbon capture and economic trait variations in subtropical bamboo forest

Jun Sun, Jinlong Li, Kohei Koyama, Dandan Hu, Quanlin Zhong, Dongliang Cheng*

*** Correspondence:** Corresponding Author: chengdl02@aliyun.com

1 Supplementary Tables

Table S1 The data of functional traits of five bamboos leaves along the different altitude gradients in Wuyi mountain.

Alititude(m)	W/L	Leaf N ($g\cdot kg^{-1}$)	Leaf P ($g\cdot kg^{-1}$)	LMA ($g\cdot cm^{-2}$)	P_n ($\mu mol\cdot m^{-2}\cdot s^{-1}$)
840	0.16	26.08	1.63	49.71	9.31
840	0.17	23.57	1.49	50.11	10.08
840	0.15	26.01	1.74	54.13	9.17
840	0.16	29.17	1.84	49.27	8.52
840	0.15	26.75	1.76	52.54	8.18
840	0.14	24.57	1.67	53.87	9.45
840	0.17	26.68	1.89	55.44	8.71
840	0.16	25.56	1.64	54.86	8.29
840	0.17	28.21	1.73	47.86	11.84
1040	0.13	29.16	1.98	52.09	9.46
1040	0.16	26.94	1.71	53.85	9.85
1040	0.14	26.10	1.68	53.24	9.52
1040	0.17	31.09	1.83	47.14	11.75
1040	0.14	25.46	1.65	48.88	8.92
1040	0.14	29.31	1.81	57.64	10.51
1040	0.15	21.41	1.22	48.13	7.56
1040	0.12	19.50	1.07	48.20	8.57
1040	0.16	22.80	1.69	55.65	8.55
1240	0.20	29.61	1.67	51.60	8.42
1240	0.17	27.59	1.46	55.75	9.22
1240	0.15	27.60	1.33	51.88	9.26
1240	0.15	29.42	1.29	48.45	11.77
1240	0.15	26.82	1.43	49.07	8.41
1240	0.15	32.49	1.84	51.66	9.65
1240	0.17	28.38	1.46	47.69	8.27
1240	0.14	25.53	1.22	50.16	9.07

1240	0.14	27.33	1.32	43.27	9.18
1100	0.12	23.05	1.14	51.09	5.16
1100	0.14	24.10	1.15	51.49	5.10
1100	0.11	23.94	1.12	50.87	5.18
1100	0.11	26.53	1.32	50.75	5.90
1100	0.12	28.86	1.32	53.09	6.13
1100	0.10	28.45	1.32	50.85	6.55
1100	0.11	27.61	1.13	50.77	7.72
1100	0.12	27.30	1.18	48.50	5.73
1100	0.13	27.84	1.17	49.01	5.48
1200	0.12	19.36	1.04	45.28	5.35
1200	0.11	19.64	1.05	45.62	5.54
1200	0.12	19.32	1.02	46.50	4.15
1200	0.12	22.46	1.09	45.58	7.77
1200	0.12	22.07	1.16	49.45	6.27
1200	0.12	21.94	1.15	46.55	4.34
1200	0.12	20.84	1.08	38.44	5.74
1200	0.13	20.65	1.07	39.13	5.81
1200	0.11	20.40	1.06	38.79	6.13
1400	0.11	21.04	1.06	41.85	5.45
1400	0.11	21.05	1.06	42.08	8.47
1400	0.10	21.22	1.10	42.24	6.55
1400	0.12	22.65	1.04	46.81	5.92
1400	0.11	22.46	1.05	50.44	5.61
1400	0.11	22.11	1.02	49.57	8.34
1400	0.12	20.76	0.99	43.87	5.42
1400	0.12	21.03	1.02	46.34	6.80
1400	0.10	20.99	1.12	45.67	7.59
1041	0.19	17.92	1.07	59.55	5.50
1041	0.18	19.91	1.24	59.80	5.50
1041	0.18	18.84	1.20	59.75	5.88
1041	0.18	19.13	1.32	52.48	5.25
1041	0.19	18.69	1.20	53.05	4.55
1041	0.17	18.72	1.23	60.01	4.64
1041	0.19	19.03	1.28	51.97	6.41
1041	0.19	18.89	1.28	51.98	5.87
1041	0.20	21.99	1.67	53.92	5.75
1440	0.15	21.52	1.56	63.81	5.04
1440	0.14	18.77	1.32	58.54	7.67
1440	0.18	21.45	1.80	57.69	9.50
1440	0.16	17.34	1.17	67.57	5.11
1440	0.16	17.82	1.21	56.47	7.72
1440	0.16	19.64	1.29	54.80	5.55
1440	0.16	19.16	1.18	60.36	6.14
1440	0.16	19.07	1.24	55.98	4.49
1440	0.15	18.82	1.20	56.52	7.91
1840	0.21	25.38	1.63	57.07	9.40
1840	0.20	22.05	1.50	62.45	6.32
1840	0.18	22.21	1.58	55.71	5.97
1840	0.17	25.06	1.60	55.61	5.77

1840	0.18	25.70	1.59	58.33	5.83
1840	0.18	27.51	1.83	56.55	8.74
1840	0.21	25.03	1.67	58.63	7.53
1840	0.20	23.03	1.53	61.21	6.08
1840	0.19	19.70	1.50	64.51	6.37
1740	0.10	22.91	1.18	53.88	5.87
1740	0.10	24.35	1.28	58.04	5.86
1740	0.11	22.86	1.14	52.01	6.18
1740	0.11	20.91	1.10	50.40	6.10
1740	0.11	22.19	1.16	55.48	9.11
1740	0.10	23.59	1.22	55.32	5.02
1740	0.11	22.27	1.23	52.47	9.13
1740	0.11	23.34	1.27	51.56	9.59
1740	0.11	19.89	1.06	60.09	4.78
2100	0.20	27.89	1.70	43.77	10.24
2100	0.17	26.21	1.55	41.60	7.75
2100	0.18	22.72	1.45	40.16	9.70
2100	0.18	30.49	1.65	41.69	9.74
2100	0.17	25.54	1.51	43.94	9.97
2100	0.18	25.92	1.65	40.70	7.86
2100	0.18	21.26	1.43	44.97	7.46
2100	0.18	22.57	1.56	42.10	8.51
2100	0.16	20.36	1.30	39.48	7.13

Note： The ratio of leaf width to length (W/L), leaf mass per area (LMA), photosynthesis rates (P_n), leaf nitrogen concentration (Leaf N), leaf phosphorus concentration (Leaf P).
